# On the Power and Legitimacy of Follow-Up Testing

**DOI:** 10.1177/03010066221081204

**Published:** 2022-03-14

**Authors:** Christopher Tyler

**Affiliations:** City University of London, Northampton Square, London, UK

In the present era of big data analysis, there is a general sense that selective data reporting, ie, cherry-picking, data-peeking, or p-hacking in any form, is wrong and leads to false or misleading conclusions (Simmons, Nelson & Simonsohn, 2011; Harvey & Liu, 2021). Faced with a large dataset with numerous testable results, it is very tempting to seize on those that register as significant at the usual criterion level and develop a rationale to account for them. On the other hand, it is widely recognized that this should not be done without correcting the criterion level for the number of tests being applied. This prohibition is generally extended to both preliminary data analysis as a study goes along, and post hoc sampling increases when a result tests as marginally significant, by adding a few extra cases to increase the sample size. Here, I argue that, to the contrary, both techniques when properly conceived are legitimate and powerful means to enhance the efficiency of scientific, and particularly medical, studies.

The preliminary data analysis issue is the more familiar one. Suppose you are conducting a large and expensive clinical trial with a randomized control design. Is it legitimate to perform preliminary tests along the way to see whether the results are significant enough for the study to be worth continuing? The answer is “No” if you use the *same* significance criterion as for the completed study, but “Yes”, if you *correct* the significance level for the number of tests conducted. The correction can be implemented according to an established method, such as the Bonferroni (1936) correction for false-positive rate or the more relaxed test for false discovery rate (Benjamini & Hochberg, 1995).

However, the preliminary analysis comes at a cost, which is that the ultimate significance criterion is higher at the completion of the study than it would have been without the preliminary analysis. For example, if the nominal false-positive criterion is *P* = .05 (z-score of 1.96, one-tailed), the cost for one preliminary assessment is that the Bonferroni correction for the final criterion would be *P* = .025 (one-tailed z-score of 2.3). For a large-scale clinical trial, however, it would seem well worthwhile to run such a check halfway through the study for this nominal cost of 17% in terms of effect size. If the results are already significant at this halfway point, there is little need to run the rest of the study. Extending the logic to continual assessment for each new participant along the way, however, would make the cost of correcting for this number of extra tests prohibitively high and should generally be avoided.

Another key issue, however, is the technique of post hoc sampling of results from large-scale data analyses such as occur in genetic studies, functional magnetic resonance imaging (fMRI), or large-scale correlation or functional connectivity analyses. The procedure known as “p-hacking” consists of overviewing the results of large numbers of statistical tests on such data and drawing conclusions from those that exceed the significance criterion, *without* correction for large number of test applications (Head et al., 2015). Conversely, applying the Bonferroni correction for, say, a million tests (which could easily occur in an fMRI connectivity analysis to and from half-a-dozen regions of interest, or regions of interest, to the rest of the voxels in the brain) would nearly triple the z-score required to reach significance (from 1.96 to 5.3), which is a high cost to pay for proper adjustment to this degree of multiple test application.

Here one should draw a profound distinction between the *illegitimate* procedure of *uncorrected* p-hacking on a completed dataset and the *valid* procedure of *post hoc sample incrementing* to bolster near-significant results. Suppose you have conducted a randomized intervention study on 10 cases and achieved a significance level of *P* = .06, just short of the required level of *P* < .05. Is it legitimate to add one more case to the sample to see if it would boost the significance to reach the criterion level? This tempting approach is widely regarded as illegitimate massaging of the dataset to manufacture falsely significant results, but this verdict does not stand up to a detailed analysis. It is in fact a perfectly legitimate approach to unearthing true effects near the margin of significance for a particular sample.

There are two hypotheses as to the underlying distribution giving rise to such a marginally significant result. One is that it was an outlier result from a null distribution with zero effect size relative to the control. The other is that it was representative sample with a legitimate effect that just missed the specified significance criterion due to an insufficient sample size.

Suppose you have tested 10 cases and obtained a *P* value of 0.06 (two-tailed, ie .03 area in each tail). Is this close enough to the *P* < .05 significance criterion to motivate continued testing to see if the p value would improve? Consider the 11th case. On the *null* hypothesis, the expected *P* value for this case is .5, implying a strong likelihood that the combined *P* value should increase (be degraded) rather than decrease (improve). If this extra participant’s data are to move the needle of the group as a whole from .06 to below the *P* < .05 criterion, its individual value must be *well below* even this .05 criterion level. And, moreover, the criterion has to be corrected to *P* < .025 (Bonferroni, 1936) to account for the use of an additional test. In fact, to reach this criterion, the individual significance level for the one extra participant would need to be *P* = .000004 (or .0003 each for two new participants.) (These *P* values may seem extreme, but they only correspond to effect z-scores of about 5 and 3, respectively, which are readily obtainable in psychophysical and brain imaging results.) So this analysis shows that, while adding one or two extra participants is highly *unlikely* to improve the outcome on the null hypothesis, if the outcome does improve to the requisite degree there is an extremely strong likelihood that there must have been a *true effect* in operation across the sample. On average, adding one or two extra participants will just dilute the result if there is no underlying effect, but should inspire full confidence in the presence of an effect if the significance does, in fact, improve.

**Figure 1.**
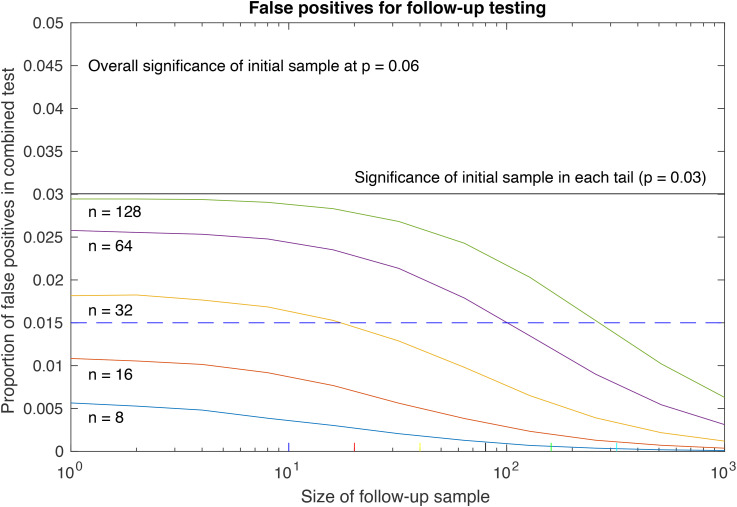
Plots of the proportional of false positives in a combined initial + follow-up sample, for initial samples of n = 10 to 160, at an initial significance level of *P* = .06 (ie 6% false positives), plotted as a function of the size of the follow-up sample. Note that the combined false-positive rate is always lower than the original false-positive rate (horizontal black line), and is reduced below the corrected significance criterion for a second test (blue dashed line) for small initial samples.

For completeness, one can calculate general results for the probability of generating false positives as a function of the size of the (second) follow-up sample, assuming the null hypothesis (Figure 1). The larger is the follow-up sample, the lower is the probability of it generating a false-positive result, scaled by the size of the initial sample. The important point to note is that the proportion of false positives generated by the follow-up procedure is always *less* than the false-positive rate for the initial sample (horizontal line at 0.03, corresponding to the boundary of the false-positive region for each tail of the two-tailed distribution).

The region of greatest interest is where both the initial sample and the follow-up sample are small (lower left corner of Figure 1), where the inclusion of the follow-up test produces a *large reduction* in the false-positive rate. When the initial sample is small (eg 8 or 16), this reduction extends to below the corrected significance criterion for a second test (ie the Bonferroni correction from *P* < .025 to *P* < .0125 in each tail, horizontal blue dashed line in Figure 1) even for one extra participant. Such a reduction implies that the inclusion of one or a few extra participants radically decreases the false-positive rate in small initial samples that initially exceeded the two-tailed *P* < .5 criterion (at *P* = .06 overall, in these examples).

This analysis shows that it is *always advantageous* to run follow-up tests for an initial sample that was close to significance, given that the appropriate correction for multiple tests is applied. This is a legitimate strategy that is inappropriately bundled under the pejorative term “p-hacking”. It is not the same as the illegitimate procedures of either running multiple tests *without* applying the appropriate statistical correction, or the specific form of that approach consisting of increasing the sample size until a significant result is obtained, which again implies the application of multiple tests without correction of the false-positive rate.

In overview, this analysis contradicts that of Simmons, Nelson and Simonsohn (2011), who present simulation data showing that extra samples beyond an original sampling of either 10 or 20 samples tend to *increase* the false-positive rate under the null hypothesis (based on purely random sampling variations). This is an obvious result given a fixed significance criterion, and the obvious solution is to correct the false-positive rate for the number of extra tests required at each step, as in the standard Bonferroni correction applied here. Bizarrely, however, these authors reject this standard approach on the grounds that it opens up the testing procedure to uncontrolled degrees of freedom by sloppy investigators. They don’t seem to recognize that the rigorous specification of the procedure through the appropriate Bonferroni correction completely corrects for the uncontrolled bias they are complaining about, as long as it is fully specified and meticulously followed.

As a result, the same authors reject the use of small samples, arbitrarily recommending a sample size of 20 participants for any psychological study, on the grounds that investigators are too slapdash to handle smaller samples with appropriate rigor. Application of such recommendations would summarily reject a large proportion of perceptual studies, which rarely extend to study samples of this size, typically preferring to obtain more extensive datasets across many independent variables for smaller study samples. This strategy has long been accepted in perception studies, but tends to encounter resistance from reviewers familiar with the different demand characteristics of studies in the cognitive sciences, which are oriented toward large study samples for restricted experimental designs with as small as 2 × 2 experimental conditions. The goal of this editorial is thus to provide the rigorous statistical rationale for a flexible sampling approach to studies using small sample sizes, particularly when it is difficult to recruit larger numbers of appropriate participants or when extensive data are obtained from each one. In these cases, appropriate correction for the number of tests applied, such as by the Bonferroni or Akaike (1974) criteria for the adjustment of the significance level, can provide the full correction for unwarranted false positives due to chance fluctuations.

Recently, Hessels and Hooge (2021) also critiqued the current emphasis on over-formalized hypothesis testing, which they oppose in favor of a more nuanced approach to science that gives full reign to the power of exploratory research to generate ideas about how to develop relevant hypotheses. This advocacy of exploratory procedures is consonant with the present argument in support of properly structured exploratory analyses. It should be noted, however, that they may have overstated the rigidity of hypothesis development from existing theories, since if a result disconfirms the predictions from all existing theories, the creative development of a novel theory or extension to the best current theory is the essential next step, and can in principle proceed on the basis of prior data without further exploratory research.
